# FIB-3 index and serum CK-18F level for monitoring suspected progression to metabolic dysfunction-associated steatohepatitis in biologic-controlled psoriasis: Two case reports and literature review on noninvasive markers

**DOI:** 10.1016/j.jdcr.2026.07.068

**Published:** 2026-08-18

**Authors:** Chikako Suzuki, Tomomitsu Miyagaki, Ken Go, Takafumi Kadono, Hidenori Watabe

**Affiliations:** a Department of Dermatology, St. Marianna University School of Medicine, Kawasaki, Japan; b Department of Dermatology, St. Marianna University Yokohama Seibu Hospital, Yokohama, Japan

**Keywords:** cytokeratin-18 fragment, FIB-3 index, liver fibrosis biomarkers, MASH, MASLD, psoriasis

## Introduction

Psoriasis is a chronic systemic inflammatory disease frequently associated with obesity and metabolic dysfunction–associated steatotic liver disease (MASLD). In 2023, the term “MASLD” was introduced to provide a pathophysiology-based definition, replacing “non-alcoholic fatty liver disease”.^1^ This framework emphasizes cardiometabolic risk factors in liver disease progression. MASLD is defined as hepatic steatosis detected by imaging studies in the presence of at least 1 cardiometabolic risk factor and in the absence of significant alcohol intake or other competing causes of steatosis.^1^ A recent meta-analysis demonstrated an increased risk of fatty liver disease in patients with psoriasis.^2^ Although MASLD often parallels psoriasis severity, it may progress independently. Metabolic dysfunction–associated steatohepatitis (MASH), a progressive form of MASLD defined as steatosis with hepatocellular inflammation and ballooning, confirmed by liver biopsy,^1^ is associated with advancing fibrosis and increased adverse outcomes. While biopsy is the diagnostic gold standard, several non-invasive markers have been proposed, including the Fibrosis (FIB)-4 index, FIB-3 index, and serum cytokeratin-18 fragment (CK-18F) levels.^3^, ^4^, ^5^ We report 2 psoriasis cases with suspected progression from MASLD to MASH, despite biologic-induced remission.

## Case report

**Fig 1:**
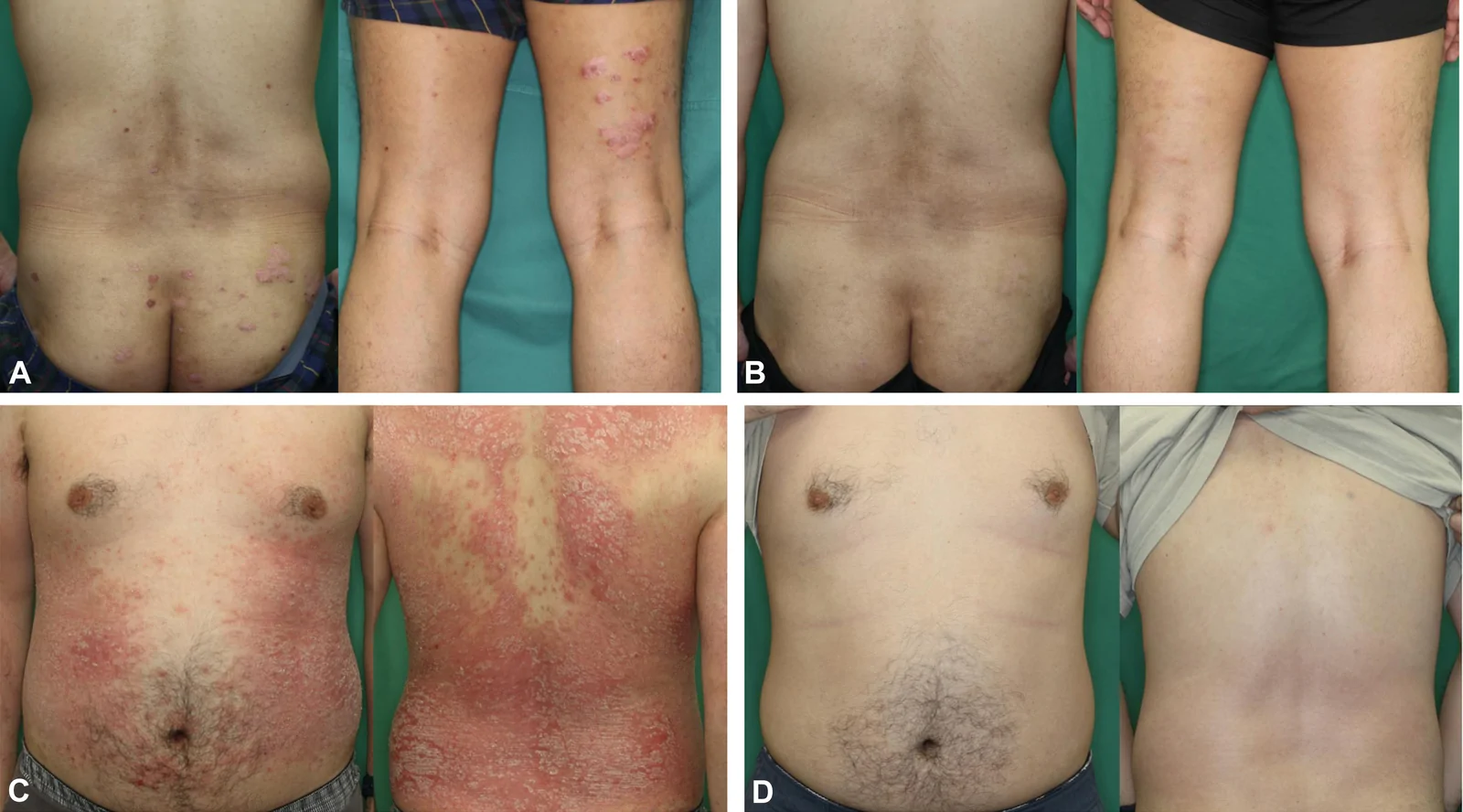
Scaly infiltrated erythematous plaques on the back and left leg before **(A)** and 12 weeks after **(B)** the administration of ixekizumab in patient 1. Infiltrated erythematous plaques on the back and left leg before **(C)** and 16 weeks after **(D)** the administration of risankizumab in patient 2.

Case 1 was a 46-year-old man with worsening psoriasis refractory to apremilast. His medical history included type 2 diabetes mellitus and dyslipidemia. At presentation, his body weight was 85 kg (body mass index [BMI] 28.4 kg/m^2^), and his Psoriasis Area and Severity Index (PASI) score was 7.6 (Fig 1, *A*). Laboratory tests revealed elevated liver enzymes (aspartate aminotransferase [AST] 52 U/L, alanine aminotransferase [ALT] 98 U/L). The FIB-4 index was 1.42 and the FIB-3 index was 2.42. Abdominal computed tomography (CT) demonstrated hepatic steatosis, and MASLD was diagnosed.^1^ Ixekizumab achieved complete skin clearance by week 12 (Fig 1, *B*). Over 5 years, his weight initially decreased to 78 kg; however, it subsequently rebounded to 85 kg. Liver enzymes increased (AST 77 U/L, ALT 117 U/L) paralleling this weight regain, with FIB-4 and FIB-3 indices rising to 2.09 and 4.14, respectively. Serum CK-18F level was markedly elevated at 874 U/L (reference range <260 U/L). Ultrasonography showed a FibroScan-AST score of 0.694, exceeding the cutoff of 0.67 for high risk of MASH,^6^ leading to a clinical diagnosis of suspected MASH.

Case 2 was a 33-year-old man referred for an inadequate response to topical therapy. At presentation, his body weight was 86 kg (BMI 28.4 kg/m^2^), and his PASI score was 38.6 (Fig 1, *C*). Laboratory tests showed elevated liver enzymes (AST 45 U/L, ALT 84 U/L). The FIB-4 and FIB-3 indices were 0.6 and 0.21, respectively. Abdominal CT revealed hepatic steatosis, and MASLD was diagnosed.^1^ Risankizumab induced complete clearance by week 16 (Fig 1, *D*). However, over 2 years, his body weight increased to 95 kg, accompanied by further elevations in liver enzymes (AST 116 U/L, ALT 143 U/L). The FIB-4 index mildly increased to 1.27, while the FIB-3 index markedly rose to 4.12. Serum CK-18F level was 887 U/L, suggesting potential progression to MASH. Changes in liver-associated markers in both cases are summarized in Table I.

**Table I:** The change in liver-associated markers and PASI in Case 1 and 2

	Case 1		Case 2	
	Before the biologics	5 y later	Before the biologics	2 y later
BW (kg)	85	85	86	95
PASI score	7.6	0	38.6	0
AST (U/L)	52	77	45	116
ALT (U/L)	98	117	84	143
Platelet (×10^4^/μL)	17.6	17.0	27.6	26.3
The FIB-4 index	1.42	2.09	0.6	1.27
The FIB-3 index	2.42	4.14	0.21	4.12
CK-18F (U/L)	-	874	-	887

*ALT*, Alanine aminotransferase; *AST*, aspartate aminotransferase; *BW*, body weight; *CK-18F*, cytokeratin-18 fragment; *FIB-3*, Fibrosis-3; *FIB-4*, Fibrosis-4; *PASI*, Psoriasis Area and Severity Index.

## Discussion

Our cases had CT-confirmed hepatic steatosis and high BMI. Serological markers for viral or autoimmune hepatitis were negative. Furthermore, no excessive alcohol intake or newly administered hepatotoxic drugs were reported during the clinical course. Consequently, both were clinically diagnosed as MASLD with potential progression to MASH. For follow-up, we used 3 noninvasive markers to assess MASLD/MASH. The FIB-4 index is calculated based on AST, ALT, platelet count, and age.^5^ Patients are generally classified as high risk (≥2.67), intermediate risk (1.30-2.67), or low risk (<1.30) for liver fibrosis.^7^ However, as age is included in the formula, the need for age-specific cutoffs has been discussed, which can make interpretation more complex. The FIB-3 index is a recently proposed marker designed to exclude the influence of age and is calculated based on AST, ALT, and platelet count.^3^ Its predictive accuracy for severe liver fibrosis is comparable to that of the FIB-4 index among MASLD patients aged <60 years and superior in those aged ≥60 years.^4^ Although its cutoff value remains under discussion, a FIB-3 index ≥3.42 suggests severe liver fibrosis in Asian MASLD patients.^3^ In our cases, following elevation of liver enzymes, the FIB-3 index markedly increased and reclassified both cases as high risk, whereas the FIB-4 index continued to indicate low or intermediate risk. These findings suggest that the FIB-3 index might serve as a useful complementary marker for predicting severe liver fibrosis in psoriasis patients with MASLD.

Additionally, we measured serum levels of CK-18F, a fragment of cytokeratin-18 released into the circulation during hepatocyte apoptosis, after the marked increase in the FIB-3 index. Serum CK-18F levels were elevated in patients with MASH compared with those with MASLD, and a cutoff of ≥750 U/L has been reported to distinguish MASH from MASLD.^5^ In our cases, CK-18F levels exceeded the cutoff, further supporting potential progression to MASH.

Another important point in our cases is that MASLD progressed to MASH even when cutaneous inflammation is adequately controlled. A recent systematic review revealed that anti-IL-17 and IL-23 inhibitors improved liver-related scores associated with MASLD in approximately half of the studies, while exhibiting a neutral effect in the remaining studies,^8^ suggesting beneficial effects of biologics on MASLD. On the other hand, a retrospective study from Mexico reported a significantly higher prevalence of MASLD in biologic-treated psoriasis patients than in non-biologic-treated patients.^9^ Larger, well-designed studies are needed to determine whether biologics for psoriasis can prevent the progression of MASLD to MASH. Interestingly, in our cases, weight gain or rebound preceded deterioration of liver function, indicating that obesity-related metabolic dysfunction may have been the primary driver of hepatocellular injury.^10^^,^^11^

A notable limitation of this report is the lack of liver biopsy and baseline CK-18F measurements. Consequently, our findings should be interpreted as preliminary clinical observations that warrant further validation in larger, prospective studies.

In conclusion, adequate control of cutaneous symptoms with biologics does not necessarily eliminate the risk of MASLD progression in patients with psoriasis. Continuous monitoring of body weight and liver function using several non-invasive markers is warranted despite well-controlled skin disease.

## Conflicts of interest

None disclosed.

## References

[1] Rinella M.E., Lazarus J.V., Ratziu V. (2023). A multisociety Delphi consensus statement on new fatty liver disease nomenclature. Hepatology.

[2] Bellinato F., Gisondi P., Mantovani A., Girolomoni G., Targher G. (2022). Risk of non-alcoholic fatty liver disease in patients with chronic plaque psoriasis: an updated systematic review and meta-analysis of observational studies. J Endocrinol Invest.

[3] Kariyama K., Kawanaka M., Nouso K. (2022). Fibrosis-3 index: a new score to predict liver fibrosis in patients with nonalcoholic fatty liver disease without age as a factor. Gastro Hep Adv.

[4] Nouso K., Kawanaka M., Fujii H. (2024). Validation study of age-independent fibrosis score (fibrosis-3 index) in patients with metabolic dysfunction-associated steatotic liver disease. Hepatol Res.

[5] Tadokoro T., Kawanaka M., Takahashi H. (2025). A noninvasive method of diagnosing metabolic dysfunction-associated steatohepatitis using cytokeratin-18 fragment and FIB-3 index. Diagnostics (Basel).

[6] Newsome P.N., Sasso M., Deeks J.J. (2020). FibroScan-AST (FAST) score for the non-invasive identification of patients with non-alcoholic steatohepatitis with significant activity and fibrosis: a prospective derivation and global validation study. Lancet Gastroenterol Hepatol.

[7] Shah A.G., Lydecker A., Murray K., Tetri B.N., Contos M.J., Sanyal A.J., Nash Clinical Research Network (2009). Comparison of noninvasive markers of fibrosis in patients with nonalcoholic fatty liver disease. Clin Gastroenterol Hepatol.

[8] González Fernández J., Prieto-Torres L., Ara Martín M., Martínez-Domínguez S.J. (2025). MASLD and liver fibrosis in patients with psoriasis receiving IL-17 or IL-23 inhibitors: a systematic review. Ther Adv Gastroenterol.

[9] Armijo-Borjon G., Miranda-Aguirre A.I., Garza-Silva A. (2025). Biologic therapy for psoriasis is associated with the development of metabolic dysfunction-associated steatotic liver disease (MASLD). A study on the association of cardiometabolic conditions with psoriasis treatment. Arch Dermatol Res.

[10] Saito T., Tsuchishima M., Tsutsumi M., George J. (2024). Molecular pathogenesis of metabolic dysfunction-associated steatotic liver disease, steatohepatitis, hepatic fibrosis and liver cirrhosis. J Cell Mol Med.

[11] Nakanishi N., Hashimoto Y., Okamura T. (2021). A weight regain of 1.5 kg or more and lack of exercise are associated with nonalcoholic fatty liver disease recurrence in men. Sci Rep.

